# Freehand and Video-Rate All-Optical Ultrasound Imaging

**DOI:** 10.1016/j.ultras.2021.106514

**Published:** 2021-07-12

**Authors:** Erwin J Alles, Eleanor C Mackle, Sacha Noimark, Edward Z Zhang, Paul C Beard, Adrien E Desjardins

**Affiliations:** a Department of Medical Physics & Biomedical Engineering, University College London, Malet Place Engineering Building, London, WC1E 6BT, United Kingdom; b Wellcome/EPSRC Centre for Interventional and Surgical Sciences, University College London, Charles Bell House, 43-45 Foley Street, London, W1W 7TS, United Kingdom

**Keywords:** All-optical ultrasound imaging, laser generated ultrasound, handheld imaging probe, real-time imaging, video-rate imaging, human *in vivo* imaging, 87.63.dh, 87.85.Pq, 43.38.Ar, 42.81.Wg

## Abstract

All-optical ultrasound (AOUS) imaging, which uses light to both generate and detect ultrasound, is an emerging alternative to conventional electronic ultrasound imaging. To date, AOUS imaging has been performed using paradigms that either resulted in long acquisition times or employed bench-top imaging systems that were impractical for clinical use. In this work, we present a novel AOUS imaging paradigm where scanning optics are used to rapidly synthesise an imaging aperture. This paradigm enabled the first AOUS system with a flexible, handheld imaging probe, which represents a critical step towards clinical translation. This probe, which provides video-rate imaging and a real-time display, is demonstrated with phantoms and *in vivo* human tissue.

## Graphical abstract

**Graphic Abstract:**
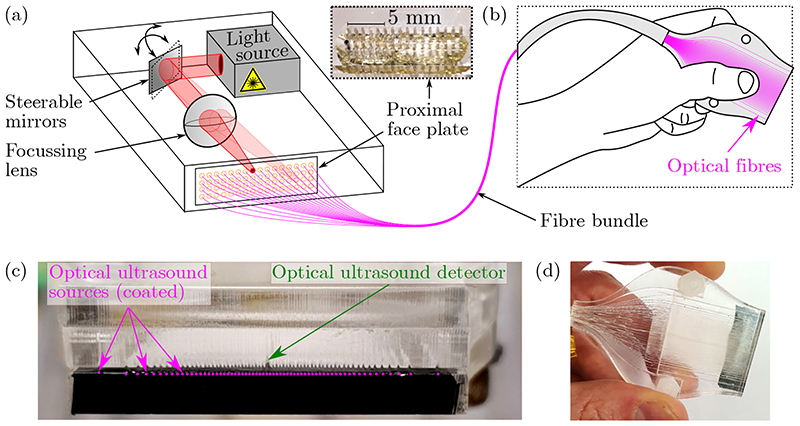
Freehand and Video-Rate All-Optical Ultrasound Imaging Erwin J Alles, Eleanor C Mackle, Sacha Noimark, Edward Z Zhang, Paul C Beard, Adrien E Desjardins

## 1 Introduction

All-optical ultrasound (AOUS) imaging, which uses light to both generate and detect ultrasound signals, is an emerging alternative to conventional electronic ultrasound technology that employs piezoelectric or capacitive transducers. With AOUS, modulated light is converted into ultrasound *via* the photoacoustic effect [1] within an optically absorbing structure [2, 3]. Contrary to most electronic ultrasound transducers, optical ultrasound sources do not rely on mechanical resonance to achieve sensitivity; as such, broad bandwidths (several tens of MHz) and high pressures (MPa range) have been routinely reported [2, 3]. Optical detection of back-scattered (“pulse-echo”) ultrasound signals [4] is typically performed using highly sensitive optically resonant structures such as Fabry-Pérot cavities [5] or ring resonators [6, 7, 8], or *via* optical interferometry [9, 10].

AOUS imaging has to date been performed using two different probe geometries. First, imaging probes comprising a single transmitter and detector have been demonstrated. Such single element imaging probes are readily miniaturised using off-the-shelf fibre-optic technology, and various highly miniaturised fibre-optic AOUS imaging probes with diameters below 1 mm have been presented [11, 7, 12, 2, 13, 14, 15, 16]. Their wide bandwidths, high sensitivities and small lateral dimensions render fibre-optic AOUS imaging probes ideally suited to biomedical imaging, and in particular to integration into minimally invasive surgical instruments. Various imaging paradigms have previously been demonstrated: a benchtop system achieved high-quality images through mechanical scanning at long time scales (minutes for 2D to hours for 3D images) [12, 2, 5]; a rotating probe achieved 2D intravascular AOUS imaging at a frame rate of 5 Hz [15]; robotic [17, 18] or manual manipulation [19] of highly directional AOUS imaging probes integrated into endoscopes enabled large field-of-view 3D imaging in a matter of minutes; and the first *in vivo* application of AOUS imaging enabled real-time guidance of a trans-septal puncture in a preclinical model [13].

Second, AOUS imaging has previously been demonstrated on the benchtop with systems that use scanning optics to arbitrarily and dynamically steer excitation light across a large monolithic optical ultrasound generator surface. This generating surface was either deposited onto the distal end of a semi-rigid coherent fibre bundle [20] (achieving 3D imaging using a probe with a diameter of 3 mm) or a planar Fabry-Pérot scanner [21], or suspended in freespace [9, 22, 23] to achieve arbitrary source aperture geometries. Using highly efficient nano-composites comprised of carbon nanotubes and polydimethylsiloxane (PDMS) [2] as optical ultrasound generating membranes, in combination with a highly sensitive fibre-optic ultrasound detector [5, 24], real-time and video-rate 2D imaging was achieved [22, 25]. However, these benchtop imaging systems were unsuitable for clinical use as either the imaging target needed to be fully submerged into a water bath, or long acquisition times were required.

In this work, we introduce a third AOUS imaging paradigm, which combines the ease and versatility in application of the fibre-optic single-element imaging probes with the rapid video-rate imaging capabilities of benchtop imaging systems that use scanning optics to synthesise a source aperture. In the presented AOUS imaging paradigm, a single fibre-optic ultrasound detector is paired with a large number of optical fibres that each act as discrete optical ultrasound sources. Due to this all-fibre design, the presented AOUS imaging probe is highly flexible, and a compact packaging allows for easy and versatile freehand imaging. In the remainder of this manuscript we will present the fabrication and acoustical characterisation of the freehand AOUS imaging probe. In addition, we will present, to the authors’ knowledge, the first freehand, video-rate and real-time 2D all-optical ultrasound imaging of both dynamic phantoms and *in vivo* human tissue.

## 2 Imaging system

**Figure 1:**
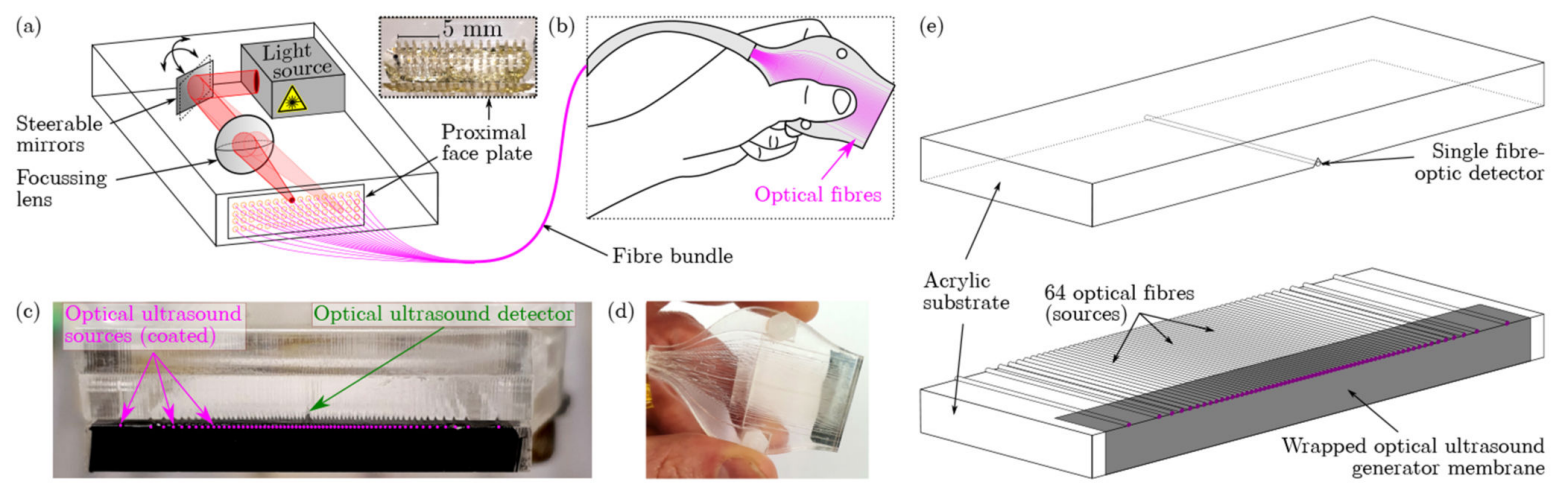
The all-optical ultrasound imaging system. (a) Schematic of the all-optical ultrasound imaging setup. Rapid scanning optics sequentially couple excitation light into 64 individual optical fibres, corresponding to 64 discrete optical ultrasound sources. The two optical paths shown correspond to the sequential excitation of two of these fibre-optic ultrasound sources. A flexible fibre bundle is used to deliver the excitation light to a photoacoustic ultrasound generating coating deposited at the distal ends of the fibres, and allows for versatile and freehand ultrasound imaging. The inset shows a photograph of the proximal face plate that houses the 64 optical fibres at a uniform spacing of 1 mm. (b) Schematic of the distal end of the imaging probe, showing the tapered and aperiodic distribution of 64 grooves (purple lines) used to position and align the fibre-optic ultrasound sources. (c) Macro photograph of the all-optical ultrasound imaging aperture. The 64 fibre-optic ultrasound sources were bonded to the surface of an acrylic substrate, which was subsequently covered with a black optical ultrasound generator membrane wrapped around the substrate edge. A second acrylic substrate houses a single fibre-optic ultrasound detector that is positioned centrally within the imaging aperture at an elevational offset of 400 μm. The approximate locations of the optical ultrasound sources are indicated with purple dots. (d) Photograph of the assembled distal end of the imaging probe. (e) Schematic (not to scale) of the distal end of the imaging probe. The optical ultrasound generator membrane is shown partially transparent to improve visibility; in reality this membrane is opaque.

To achieve freehand video-rate AOUS imaging using a handheld probe, a set of mirror galvanometers (GVSM002, Thorlabs, Germany) and a lens (focal length: 50 mm; LBF254-050-C, Thorlabs, Germany) were used to couple pulsed excitation light (wavelength: 1064 nm; pulse duration: 1.5 ns; pulse repetition rate: 1 kHz; DSS1064-Q4, Crylas, Germany) into the proximal ends of a number of discrete optical fibres. The low inertia of these galvonometers allowed for rapid switching between the discrete optical fibres at a rate of several kHz, which enabled video-rate image acquisition. The excitation light was guided to an optically absorbing coating deposited at the distal ends of the fibres, where it was converted into ultrasound *via* the photoacoustic effect. Thus, by successively scanning the excitation light across the proximal ends of the fibres, a distally-located ultrasound source aperture could be rapidly scanned. Back-scattered ultrasound signals were detected using a single, highly sensitive fibre-optic sensor comprising a planoconcave Fabry-Pérot cavity [5, 24], and the resulting signals were digitised, processed, reconstructed into images, and displayed in real-time. The experimental setup is schematically shown in Fig. 1(a).

### 2.1 Imaging probe

A compact, handheld AOUS imaging probe (Fig. 1(b-c)) was fabricated with dimensions (40 mm × 40 mm × 10 mm) that were consistent with those of compact electronic imaging probes widely used in clinical practice. This imaging probe was composed of two custom laser-cut acrylic substrates. One substrate housed a single fibre-optic ultrasound detector centrally within the imaging aperture at an elevational (“out-of-plane”) offset of 400 μm; the other substrate housed 64 bare optical fibres (core diameter: 200 μm; flat-cleaved; FT200UMT, Thorlabs, Germany) corresponding to the 64 fibre-optic ultrasound sources. These fibres were distributed across a linear aperture with a width of 25 mm, and arranged in a non-periodic pattern that was previously demonstrated to minimise image artefacts associated with side and grating lobes [25]. Both the fibre-optic ultrasound sources and the detector were bonded to the acrylic substrates using UV-curing adhesive (NOA68, Norland Products, NJ, USA).

A nano-composite film comprising multi-walled carbon nanotubes and PDMS was doctor-bladed to obtain a membrane measuring 49 ± 6 μm in thickness [22]. This membrane, which efficiently converted excitation light into ultrasound, was wrapped around the edges of the substrate housing the fibre-optic ultrasound sources (Fig. 1(d)) and affixed to the fibre tips and substrate using uncured PDMS (MED-1000, Polymer Systems Technology, UK, diluted with xylene). The two substrates were assembled into a compact imaging probe that collected the optical fibres into a flexible fibre bundle (length: *ca*. 1.5 m), and thus allowed for versatile and easy manual handling and operation.

### 2.2 Acoustical characterisation

The acoustical performance of the freehand AOUS imaging probe was assessed through a series of acoustic field scans. A calibrated needle hydrophone (calibrated bandwidth: 1 – 30 MHz; diameter: 75 μm; Precision Acoustics, UK) was placed at a distance of 1.6 mm from the acrylic substrate, and scanned across a plane orthogonal to the optical fibres using a set of orthogonal motorised stages (step size: 50 μm; area: 30 mm × 2 mm; MTS50/M-Z8 + TDC001, Thorlabs, Germany). For each needle hydrophone position, 64 ultrasound signals generated by the 64 optical ultrasound sources were recorded and digitised (sampling frequency: 250 MHz; bit-depth: 14 bits; M4i.4420-x8, Spectrum, Germany). For these acoustic field measurements, an optical pulse energy of 8 μJ was used.

### 2.3 Signal processing

Back-scattered ultrasound signals were detected optically using a highly sensitive fibre-optic ultrasound detector comprising a plano-concave Fabry-Pérot cavity at its tip. Impinging ultrasound waves modulated the thickness of this cavity; back-scattered ultrasound waves could hence be detected by monitoring the reflectivity of the cavity. To interrogate the fibre-optic ultrasound detector, continuous-wave light (1500 – 1600 nm; 5.0 mW; Tunics T100S-HP CL, Yenista Optics, France) was continuously tuned to the wavelength corresponding to the greatest pressure sensitivity, and delivered to the ultrasound detector through a circulator (6015-3-APC, Thorlabs, Germany). The reflected light was detected using a custom photodetector, high-pass filtered (cut-off: 500 kHz) and digitised. No signal averaging was applied.

Back-scattered radio-frequency time traces recorded for each of the 64 fibre-optic ultrasound sources were collected into a single “B-scan” that was reconstructed into an image using a “Delay & Sum” algorithm (equivalent to “dynamic focussing”) [26], followed by envelope detection and log compression. The image reconstruction algorithm was implemented in parallelised fashion on a Graphical Processing Unit (GPU; Quadro P6000, Nvidia Corporation, CA, USA), and reconstruction of the current image was performed during data acquisition for the next image [25]. Through this highly parallelised approach, real-time data acquisition, processing, and visualisation was achieved at a sustained frame rate of 11 Hz.

### 2.4 Imaging scenarios

Three imaging scenarios were considered in this work. First, to assess the imaging performance, a single tungsten wire (diameter: 27 μm) was submerged in water and placed centrally within the image at an axial distance of 4 mm. This wire was placed perpendicular to the image plane and acted as a point target; this enabled the assessment of the resolution, contrast and signal-to-noise ratio of the AOUS imaging system.

Second, a phantom consisting of a tissue-mimicking material was imaged. This phantom was fabricated using 10% poly(vinyl) alcohol (PVA) cryogel, with 0.5%-wt glass spheres added to achieve physiologically realistic appearance under ultrasound imaging [27]. Embedded within this phantom was a wall-less cavity emulating a blood vessel, and this phantom was used to demonstrate the dynamic imaging of a needle insertion (needle shaft: 23g, outer diameter: 0.64 mm). For this experiment, the phantom was submerged in water and placed in direct contact with the AOUS imaging probe.

Third, the freehand AOUS imaging probe was placed on the skin of the neck to image the common carotid artery of the first author (EJA), to demonstrate both the versatility in operation and clinical relevance of the presented AOUS imaging system. In this case, the AOUS imaging probe was wrapped in a thin polyvinyl chloride film (PVC “cling film”; thickness: 150 μm) for mechanical protection, and clinical ultrasound coupling gel was applied between the imaging probe and the skin. This procedure mirrors one frequently used in clinical practice, where a surgical glove is wrapped around the imaging probe to facilitate sterilisation. For all three scenarios, ultrasound was generated using an optical pulse energy of 40 μJ.

## 3 Results

### 3.1 Acoustical performance

**Figure 2:**
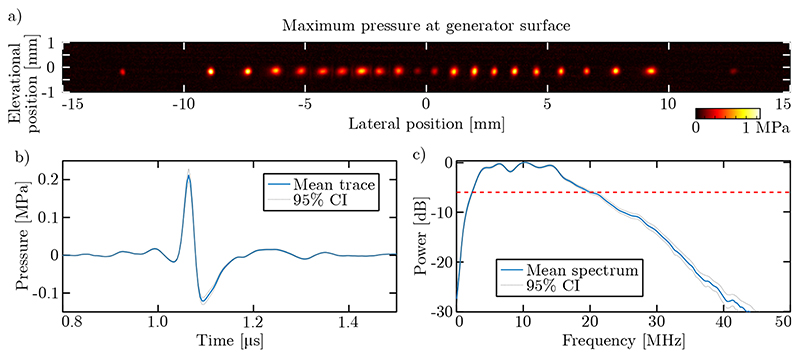
Acoustical performance of the all-optical ultrasound imaging array. (a) Composite visualisation of the maximum pressure observed at the optical ultrasound generator surface. Separate acoustic field scans were performed for each of the 64 fibreoptic ultrasound sources; for clarity the pressure field of only every third source is shown. (b) The mean and 95% confidence interval of the time traces of the pressure recorded at a distance of 1.6 mm directly in front of each of the 64 fibre-optic ultrasound sources. (c) The mean and 95% confidence interval of the power acoustic spectra of the 64 fibre-optic ultrasound sources.

Field scans revealed that the acoustical performance of the 64 optical ultrasound sources was reasonably uniform (Fig. 2). At a distance of 1.6 mm, a peak pressure across the fibre-optic ultrasound sources of 0.24 ± 0.07 MPa (mean ± standard deviation), a high centre frequency of 11.2 ± 0.9 MHz, and a wide – 6 dB bandwidth of 17.4 ± 1.7 MHz (corresponding to a fractional bandwidth of 155%) were observed. However, the fibre-optic ultrasound sources (with diameters of 200 μm) are increasingly directional for high frequencies (above *ca*. 7.5 MHz). Therefore, to avoid image reconstruction artefacts associated with a high source directionality, the B-scans in all remaining experiments were band-pass filtered (cut-off: 2 – 7 MHz).

The angular spectrum approach (ASA) [28] was used to numerically back-propagate these acoustic field scans to obtain the pressure amplitude at the optical ultrasound generating surface (Fig. 2(a)). Note that the accuracy of the ASA method is limited by the necessarily finite spatial extent of the field scan, resulting in the lower amplitudes observed at the edges of the aperture where part of the emitted acoustic ultrasound is not captured. However, this approach did confirm that the optical ultrasound sources exhibited circular symmetry, and resulted in only a weak directivity in both the lateral and elevational direction. As a result, a relatively low elevational resolution (equivalent to the imaging slice thickness) of 3.4 mm was obtained at an axial depth of 6 mm (data not shown).

### 3.2 Imaging performance

**Figure 3:**
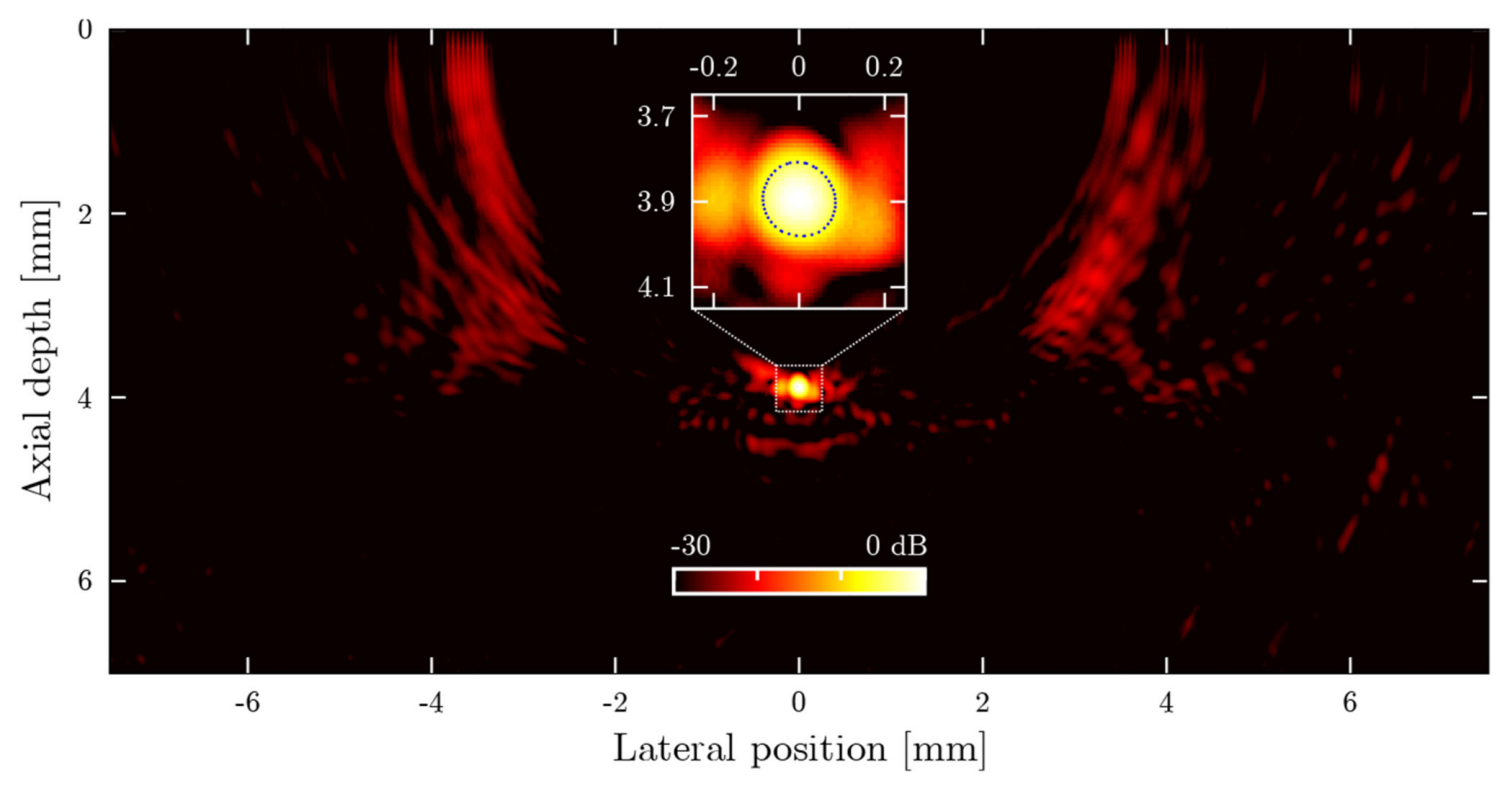
Point spread function of the all-optical ultrasound imaging system. All-optical ultrasound image of a tungsten wire (diameter: 27 μm; acting as a point target) placed centrally within the image at an axial depth of 3.9 mm. The insert shows a magnification of the image, and the –6 dB level corresponding to the spatial resolution of the imaging system is indicated by the dotted contour. The image is shown on a normalised logarithmic scale at a dynamic range of 30 dB.

AOUS images of a point-like structure (Fig. 3) located centrally within the image yielded a near-isotropic spatial resolution of 169 μm (lateral) by 173 μm (axial). Phantoms comprising multiple point-like scatterers (data not shown), as well as previously reported work [22, 25], confirm this spatial resolution is constant across the image due to the dynamic focussing applied upon image reconstruction. However, the low number of optical ultrasound sources (64) resulted in residual grating lobes (the “wing-shaped” artefacts observed at a lateral distance of approximately 3.5 mm) at an image signal-to-clutter ratio (SCR) of 21 dB, despite a high signal-to-noise ratio (SNR) of 19 dB observed in the B-scans prior to image reconstruction.

### 3.3 Dynamic imaging - phantom

**Figure 4:**
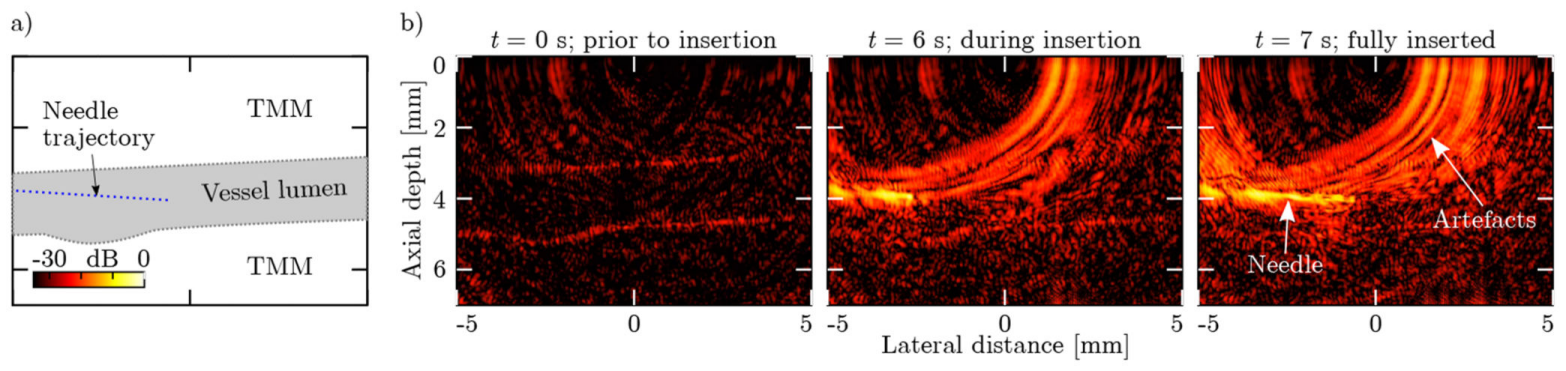
Dynamic all-optical ultrasound imaging. **(a)** Schematic of the dynamic phantom imaging experiment. An imaging phantom composed of a tissue-mimicking material (TMM) was placed in direct contact with the the all-optical ultrasound (AOUS) imaging probe, and aligned with the long axis of a wall-less structure (mimicking a blood vessel) contained within the phantom. This phantom was continuously imaged whilst a needle was manually inserted into and retracted from the vessel lumen along the trajectory indicated by the blue dotted line. **(b)** AOUS images obtained at different time points. In the absence of a needle (*t* = 0 s), the vessel walls are clearly visualised. The presence of a needle, however, introduced strong “wing-shaped” artefacts that partially obscured the phantom. All images are shown on the same normalised logarithmic scale at a dynamic range of 35 dB. A video of the longitudinal imaging experiment can be found in Supplementary Movie 1.

Dynamic imaging of a tissue-mimicking phantom confirmed that the handheld AOUS imaging probe achieved sufficient sensitivity to clearly visualise the walls of an emulated blood vessel, and in addition was capable of monitoring the placement of a needle in real-time and at video-rate (Fig. 4 and Supplementary Movie 1). However, due to the grating lobe artefacts discussed previously in section 3.2, a limited contrast of 13 dB was observed. In addition, the high echogenicity of and acoustic ringing within the needle gave rise to strong image artefacts that partially obscured the signal from the vessel phantom.

### 3.4 Dynamic imaging - in vivo

**Figure 5:**
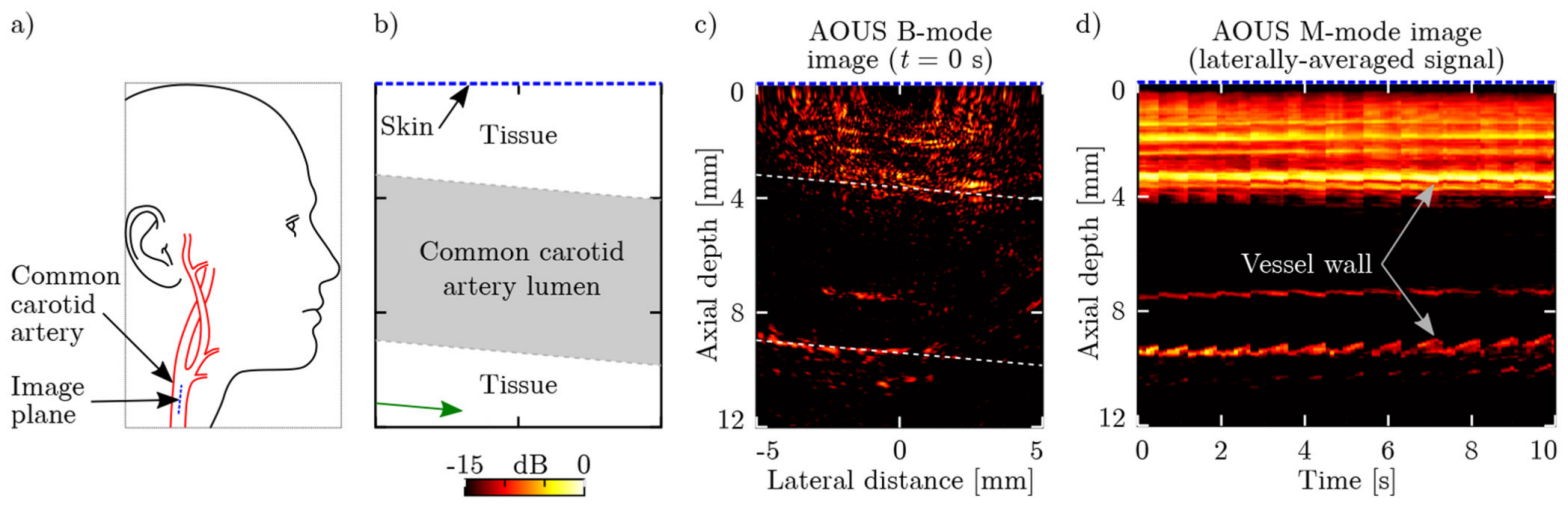
*in vivo* all-optical ultrasound imaging. **(a)** Schematic indicating the location of the image plane. The all-optical ultrasound (AOUS) imaging probe was placed directly on and orthogonal to the skin of the neck of a human volunteer, and positioned longitudinally along the common carotid artery. The imaging plane was positioned centrally through the artery lumen. **(b)** Schematic of the 2D AOUS image, with the skin located at the top. **(c)** Real-time B-mode AOUS imaging was performed over a 10 s interval at a frame rate of 11 Hz. The image of a human common carotid artery at time *t* = 0 s is shown. The artery walls are indicated by the dashed lines, and the image is displayed on a normalised logarithmic scale at a dynamic range of 15 dB. **(d)** For each B-mode frame, the image amplitude was laterally averaged at each axial depth along the slanted direction indicated by the green arrow in panel (b). The resulting M-mode image, comprising concatenated laterally-averaged signals, enabled clear visualisation of the expansion and contraction of the artery wall due to pulsatile blood flow. A video of this *in vivo* AOUS imaging experiment can be found in Supplementary Movie 2.

Real-time and video-rate AOUS imaging of an *in vivo* human common carotid artery confirmed that the presented AOUS imaging system was capable of clearly visualising the carotid artery, as well as dynamically monitoring the blood vessel geometry (Fig. 5 and Supplementary Movie 2). A contrast (13 dB) and artefact level were observed that were similar compared to those observed for the tissue-mimicking phantom in the absence of a needle. Based on the discontinuties observed in the motion of the distal blood vessel wall (at a depth of 9.5 mm, Fig. 5(d)), the heart rate varied between 87 BPM (at time *t* = 0 s) and 60 BPM (at *t* = 7 s). In addition, the blood vessel rapidly expanded in diameter by approximately 0.2 mm during systole (measured at the distal wall), followed by a slower relaxation during diastole. This vessel expansion was less pronounced for the proximal blood vessel wall (located at a depth of 3.5 mm) due to dampening by pressure applied through the skin by the AOUS imaging probe.

## 4 Discussion and Conclusion

In this work a novel imaging paradigm for all-optical ultrasound (AOUS) imaging is presented, where a single fibre-optic ultrasound detector was paired with a number of discrete fibre-optic ultrasound sources. These sources were sequentially excited in rapid succession through the use of scanning optics. Using off-the-shelf optical components and rapid prototyping techniques, a compact and flexible AOUS imaging probe was fabricated in-house that allowed for freehand and versatile operation. The presented system is, to the authors’ knowledge, the first freehand AOUS imaging system capable of real-time and video-rate 2D imaging, and yielded images of sufficient contrast and image quality to visualise clinically relevant structures.

The use of optical ultrasound transducers offers substantial benefits over conventional electronic transducer technology. First, AOUS sources and detectors are readily miniaturised whilst retaining a high sensitivity, and typically exhibit broad bandwidths resulting in high image resolutions. Second, off-the-shelf fibre-optic components can be employed to facilitate cost-effective probe fabrication. Third, the AOUS imaging probe presented here is comprised entirely of glass and plastic, and is hence inherently compatible with magnetic resonance imaging (MRI). In addition, an absence of frontend electronics renders AOUS imaging probe resilient against electromagnetic interference, thus enabling long cable lead lengths (tens of metres) and applications in electromagnetically harsh environments such as radio-frequency ablation [29]. Finally, by switching to optical ultrasound generating membranes exhibiting wavelength-selective absorption [14], AOUS imaging probes can be rendered transparent across a desired wavelength range to enable colocalised light delivery for use in, for instance, photoacoustic imaging [14] or photodynamic therapy.

Compared to technologically mature conventional electronic ultrasound imaging systems, the prototype AOUS imaging system presented here exhibits a lower frame rate, lower contrast, lower dynamic range, and lower elevational resolution. The elevational resolution could be improved through the addition of an acoustically focussing lens, or by employing eccentric optical wave guides to achieve eccentrically shaped optical ultrasound sources that spatially confine the emitted ultrasound to a narrow image plane [22]. The frame rate could be increased by switching to a light source with a higher pulse repetition rate (PRR); a modest PRR of 3 kHz would theoretically allow for a frame rate of 93 Hz. Improving the contrast and dynamic range, however, is less straightforward as they are limited by the image artefacts associated with the grating lobes of the imaging probe (*cf*. Fig. 3). These image artefacts result from the low number of transducers (64 sources and a single detector); thus, to decrease these artefacts, ideally a larger number of sources or detectors is used.

Increasing the number of fibre-optic ultrasound detectors is impractical due to high equipment costs and technological challenges. Increasing the number of sources within the imaging aperture would require the use of smaller optical ultrasound sources, and consequently lower pulse energies to avoid optical damage to the ultrasound generating membrane. The corresponding reduction in B-scan SNR could be offset either through the application of coded excitation [30], or by switching to eccentric optical ultrasound source geometries that (as previously demonstrated in a free-space AOUS imaging setup [22]) confine the ultrasound energy to just the image plane and thus limit SNR reduction with depth. Alternatively, advanced image reconstruction algorithms could be applied to further suppress the image artefacts at the expense of an increase in computational complexity, such as Delay, Multiply and Sum [31], Short-Lag Spatial Coherence [32], or deep learning approaches [33].

The imaging paradigm presented in this work allowed for, to the authors’ knowledge, the first video-rate, *in vivo* AOUS imaging of human tissue at sufficient contrast to visualise clinically relevant targets. The compact and flexible fibre-optic design of the imaging probe allowed for handheld and versatile operation. Furthermore, due to the materials used, and the absence of front-end electronics, the presented imaging probe is well-suited to concurrent multimodal applications in conjunction with, for instance, magnetic resonance imaging. The presented imaging probe and paradigm thus show great promise for future clinical applications of all-optical ultrasound imaging.

## Supplementary Material

Supplementary movie 1

Supplementary movie 2

## Highlights

**Freehand and Video-Rate All-Optical Ultrasound Imaging**

Erwin J Alles, Eleanor C Mackle, Sacha Noimark, Edward Z Zhang, Paul C Beard, Adrien E Desjardins

- A novel all-optical ultrasound imaging paradigm using a handheld probe is presented
- Video-rate and real-time all-optical ultrasound imaging are demonstrated
- The first-on-man all-optical ultrasound imaging with a handheld probe is presented
